# Relevance of prematurity and foetal growth restriction for romantic relationships, health-risk behaviours, and socio-economic outcomes in adulthood

**DOI:** 10.1093/eurpub/ckag105

**Published:** 2026-07-14

**Authors:** Achim Fieß, Sandra Gißler, Alexander K Schuster, Jonas Tesarz, Eva Mildenberger, Elmar Brähler, Michael S Urschitz, Norbert Pfeiffer, Manfred E Beutel, Alica Hartmann, Mareike Ernst

**Affiliations:** Department of Ophthalmology, University Medical Center of the Johannes Gutenberg University Mainz, Mainz, Germany; Department of Ophthalmology, University Medical Center of the Johannes Gutenberg University Mainz, Mainz, Germany; Department of Ophthalmology, University Medical Center of the Johannes Gutenberg University Mainz, Mainz, Germany; Department of Psychosomatic Medicine and Psychotherapy, University Medical Center of the Johannes Gutenberg University Mainz, Mainz, Germany; Division of Neonatology, Department of Pediatrics, University Medical Center of the Johannes Gutenberg University, Mainz, Germany; Department of Psychosomatic Medicine and Psychotherapy, University Medical Center of the Johannes Gutenberg University Mainz, Mainz, Germany; Department of Medical Psychology and Medical Sociology, University of Leipzig Medical Center, Leipzig, Germany; Division of Paediatric Epidemiology, Institute of Medical Biostatistics, Epidemiology, and Informatics, University Medical Centre of the Johannes Gutenberg University Mainz, Mainz, Germany; Department of Ophthalmology, University Medical Center of the Johannes Gutenberg University Mainz, Mainz, Germany; Department of Psychosomatic Medicine and Psychotherapy, University Medical Center of the Johannes Gutenberg University Mainz, Mainz, Germany; Department of Ophthalmology, University Medical Center of the Johannes Gutenberg University Mainz, Mainz, Germany; Department of Psychosomatic Medicine and Psychotherapy, University Medical Center of the Johannes Gutenberg University Mainz, Mainz, Germany; Department of Clinical Psychology, Psychotherapy and Psychoanalysis, Institute of Psychology, University of Klagenfurt, Klagenfurt am Woerthersee, Austria

## Abstract

Advances in neonatal care have improved survival rates of preterm and growth-restricted infants. However, concerns remain regarding their long-term psychosocial development. This study investigated psychosocial outcomes in adults born preterm or growth-restricted. The Gutenberg Prematurity Study is a retrospective cohort study including 606 participants (326 women, 53.8%) aged 18–52 years. Participants were categorized by gestational age (GA) as extremely preterm (≤28 weeks), very preterm (29–32 weeks), moderately preterm (33–36 weeks), and term (≥37 weeks), and by birth weight percentile as small for GA (SGA), appropriate for GA, and large for GA. Logistic regression assessed associations of GA and birth weight with romantic relationships and health-risk behaviours. Socio-economic status (SES) parameters were analysed using linear regression. Extremely and very preterm participants reported fewer health-risk behaviours, particularly lower alcohol consumption [extremely preterm: odds ratio (OR) = 0.26; 95% confidence interval (CI): 0.13–0.49; *P* < 0.001]. Similarly, participants born SGA were less likely to report current alcohol consumption (OR = 0.63; 95% CI: 0.41–0.98; *P* = 0.04). A trend towards lower odds of ever having been in a romantic relationship was observed (OR = 0.35; 95% CI: 0.11–1.09; *P* = 0.07). Being born extremely or moderately preterm was associated with lower overall SES (extremely preterm: β = −2.25; 95% CI: −3.22 to −1.27; *P* < 0.001; moderately preterm: β = −1.04; 95% CI: −1.73 to −0.35; *P* = 0.003). No significant associations were found for present relationship status, relationship satisfaction, or smoking behaviour. Preterm birth and foetal growth restriction are associated with adult psychosocial outcomes, with preterm birth more strongly affecting socio-economic outcomes, underscoring the need for targeted support.

## Introduction

Prematurity, particularly very preterm birth or very low birth weight (LBW), can result in various biological disadvantages such as brain abnormalities [1], cognitive problems [2], and fine motor deficits [3]. A growing body of research documents their relevance for development and health and well-being over the whole (adult) lifespan. Nevertheless, while the biological impacts are well-documented, there is a significant gap in our awareness of the social consequences that these conditions can have up to adulthood. Prematurity and foetal growth restriction may have long-term consequences beyond medical outcomes and can extend into social domains as well. This may include the development of romantic relationships, overall well-being, emotional regulation, and health behaviours. These effects may occur alongside differences in academic achievement and later socio-economic status (SES) [4]. Importantly, these domains overlap and interact throughout development. The biopsychosocial model states that illnesses are not solely the result of biological aspects, but rather arise from interactions between physical, psychological, and social factors [5]. For example, a lower educational status may increase stress and thus contribute to difficulties in the quality of romantic relationships (conflict and affection) [6]. Similarly, health-risk behaviours such as smoking and alcohol use could negatively impact SES through various pathways, including educational attainment and employment opportunities [7, 8]. Romantic relationships could potentially influence health-risk behaviours, possibly acting as a protective factor [9]. Understanding, first, how psychosocial outcomes are impacted and, second, how they are linked, is vital for developing targeted interventions mitigating potential long-term consequences of prematurity and foetal growth restriction.

Romantic relationships are directly associated with higher subjective happiness [10] and relationship satisfaction, positively influencing mental and physical health [11]. Current studies show that individuals born preterm have greater difficulties in social interactions both in childhood [12] and in adulthood [13], than individuals born full term. Evidence also indicates that individuals born preterm or individuals born with a LBW are less likely to enter into romantic relationships [14]. A meta-analysis examined 21 studies and showed that individuals born preterm or with LBW are less likely to enter into romantic relationships [odds ratio (OR) 0.72; 95% confidence interval (CI) 0.64–0.81] or become parents (OR 0.77; 95% CI 0.65–0.91) [14]. However, most studies did not differentiate between grades of prematurity and foetal growth restriction as contributing factors. This lack of distinction makes it challenging to identify the specific impacts of each condition on social relationships.

In addition to the consequences for social relationships, prematurity and/or LBW may influence socio-economic outcomes such as education, employment, or income. In the Gutenberg Health Study, an association between LBW (<2500 g) and lower SES was observed primarily among women, whereas no significant association was found among men [15]. A Danish study extracted data from 203 283 individuals from the national registers (aged 27–29 years), showing that individuals born preterm had a lower educational level and 11% lower net income than individuals born full term. Moreover, the preterm group was more likely to live alone without children (28.8% vs 21.8%). Interpretations of these findings is limited because birth weight percentile was not included as a contributing or confounding factor in the analysis [16]. Additionally, research on socio-economic parameters has shown that adults born very preterm tend to complete fewer years of education and leave school earlier compared to their peers [17].

Besides the observed associations with socio-economic parameters, health behaviour, which is closely linked with educational attainment, SES [18], and well-being, might also differ in contrast to individuals born full term. A Dutch 19-year follow-up study examined 656 adolescents born preterm or with LBW [<32 weeks of gestational age (GA), a birth weight of 1500 g, or both] and demonstrated that the preterm or/and LBW group showed less health-risk behaviour. This included less smoking, less alcohol consumption, and lower overall lifetime drug use [19], while no distinction was made between prematurity and foetal growth restriction.

Thus, the aim of this study was to analyse the associations between different levels of preterm birth and birth weight percentile as a surrogate marker for foetal growth restriction with social relationships, health-risk behaviour, and socio-economic factors in adulthood. There are limited data in the medical literature comparing the effects of different degrees of prematurity and foetal growth restriction across GAs. This raises the question of which factor primarily drives differences across these domains and whether there are thresholds of prematurity below which social development is particularly affected. Therefore, this study aims to address this gap. Understanding how early biological factors like GA and birth weight percentile relate to adult behaviours and social relationships might help healthcare providers offer more personalized care and support, especially with a focus on modifiable factors such as health behaviour. We hypothesized that lower GA and lower birth weight percentiles would be associated with reduced likelihood of forming romantic relationships, less engagement in health-risk behaviours, and lower SES in adulthood. Moreover, we expected that lower GA would have a stronger impact than birth weight percentile on these outcomes.

## Methods

### Study population

The Gutenberg Prematurity Study (GPS) is a retrospective cohort study conducted at the University Medical Center of the Johannes Gutenberg University Mainz (UMCM), Germany. It includes participants born either preterm or full term between 1969 and 2002, who were aged 18–52 years at the time they underwent medical evaluations and completed interviews. This study combined elements of retrospective cohort analysis with prospective data collection. Utilizing birth records from 1969 onward, an invitation was sent to every second individual born preterm with a GA of 33–36 weeks, and to all those born preterm at 32 weeks of gestation or less at UMCM during the specified period. To create a control group matched for age and sex, six individuals born full term (three men, three women) from the 10th to 90th birth weight percentiles were selected for each birth month spanning from 1969 to 2002, as described in previous studies [20, 21]. Furthermore, to enable a stratified analysis of the effects of different levels of foetal growth independent of prematurity, we specifically invited participants born full term into three groups based on birth weight percentiles: small for GA (SGA; birth weight percentile <10th), appropriate for GA (AGA; 10th to 90th percentile), and large for GA (LGA; >90th percentile). Study design details are provided in Fig. S1.

Participants were categorized by their GA. Group 1 included those born at 37 + 0 weeks of gestation or later, totalling 296 participants. Group 2 comprised 137 individuals born at a GA between 33 + 0 and 36 + 6 weeks. Group 3 consisted of 116 individuals born between 29 + 0 and 32 + 6 weeks, and Group 4 included 57 participants born at or before 28 + 6 weeks. From 2019 to 2021, these participants underwent comprehensive medical evaluations and participated in personal interviews.

All participants provided written informed consent prior to joining the study, adhering to the standards of Good Clinical Practice, Good Epidemiological Practice, and the ethical guidelines of the Declaration of Helsinki. The study’s protocol and related documents received approval from the local ethics committee of the Medical Chamber of Rhineland-Palatinate, Germany (reference no. 2019-14161; original approval: 29 May 2019, most recent update: 02 April 2020).

### Data collection process

Initially, all participants enrolled in the study underwent comprehensive sociodemographic and clinical assessment via questionnaire. Subsequently, they were invited for a clinical interview and physical examination, during which the questionnaire data were confirmed and any missing details were supplemented. SES was evaluated using the multidimensional index from the Robert Koch Institute’s Federal Health Survey in Germany [22]. To determine SES, three sub-dimensions, education, occupation, and income (net equivalent income), were quantified into metric sub-scales (ranging from 1 to 7), culminating in a cumulative score [3–21, 22]. Net equalized income was calculated by dividing the total monthly household income by the weighted household size. The household weight was determined as follows: the first adult contributed 1.0 unit, each additional person aged 14 and over added 0.5 units, and each child under the age of 14 added 0.3 units. Romantic Relationships: romantic relationships were assessed using four key items: (i) ever been in a relationship (Yes); (ii) present relationship (Yes); (iii) satisfied with relationship (Yes); and (iv) having children (Yes). The item “having children” referred to self-reported parenthood, regardless of whether the children were biological or adopted. These items provide a simple, yet comprehensive overview of the participants’ relationships. Health-risk behaviours were assessed using two key items: (i) current smoking (Yes) and (ii) current alcohol consumption (≥1 glass/week) (Yes).

### Assessment of prenatal and postnatal history

Medical records at UMCM contained perinatal variables including GA (weeks), placental insufficiency, pre-eclampsia, maternal smoking during pregnancy and breastfeeding history, based on records and maternal report. These variables are presented to provide a comprehensive characterization of the study cohort. Additionally, birth weight percentiles were calculated using the methodology described by Voigt *et al.* [23]. This reference is based on a large national birth cohort of more than 2.3 million live-born singleton infants born between 1995 and 2000 in Germany. Percentiles were determined separately for sex and GA, allowing for precise adjustment of birth weight relative to GA norms.

### Statistical analysis

For dichotomous parameters, we determined both absolute and relative frequencies. For variables displaying a normal distribution, we calculated the mean and standard deviation. To evaluate differences in social outcomes across various GA groups, we conducted an analysis of variance for continuous variables and the chi-square test for categorical variables, followed by the calculation of global *P-*value.

Multivariable logistic regression analysis was used to assess the associations of different levels of prematurity and foetal growth restriction with adult social relationships and health-risk behaviour in adulthood. Independent variables comprised GA divided into three categories (GA of 33–36 weeks, 29–32 weeks, and ≤28 weeks) and birth weight percentile divided into two categories (SGA <10th percentile and LGA >90th percentile). Participants born AGA (10th to 90th percentile) served as the reference group when assessing the effects of foetal growth restriction, and participants born at term (≥37 weeks of gestation) served as the reference group when assessing the effects of GA categories. In addition, socio-economic parameters (education, occupation, income, and overall SES) were analysed using multivariable linear regression analysis. Model 1 included GA groups as the primary exposure, while model 2 additionally included birth weight percentile groups (SGA, LGA) as covariates to account for foetal growth differences. All models were adjusted for age and gender. Age was included to account for differences in participants’ birth cohorts (given the wide birth period) and age-related variation in the outcomes at the time of assessment. In additional analyses, we examined potential gender differences by including gender as a main effect and testing interaction terms between gender and the continuous gestational deficit across outcomes. Because interaction effects are often associated with lower statistical power, interaction terms with *P-*value between 0.05 and 0.10 were interpreted cautiously as suggestive trends [24]. To facilitate interpretation of significant or suggestive interaction terms, gender-specific estimates and 95% confidence intervals were derived from re-parametrized models with alternating reference categories for gender, following a previously described approach [25].

### Sensitivity analyses

To increase statistical power and verify the comparability of the results, we repeated the analysis using continuous parameters with the GA deficit (the number of weeks shorter than the standard 40-week term) and birth weight percentile. An additional sensitivity analysis for socio-economic outcomes included further adjustment for maternal education score as an indicator of parental socio-economic position. Information on maternal education was available for 257 mothers only, which reduced the statistical power of these analyses.

A further sensitivity analysis was conducted for the outcome ‘having children’, including only participants aged 35 years and older. Parenthood is strongly age-dependent and often delayed until the mid-30s in contemporary populations. Therefore, restricting the analysis to participants aged 35 or older minimizes the risk of misclassifying participants as childless simply due to their younger age, and provides a more accurate assessment of the association between prematurity and adult parenthood. Selecting 35 years as the cut-off ensures that most participants had sufficient time and opportunity to experience parenthood, thereby reducing the risk of misclassification due to age-related delays in childbearing.

This is an explorative study; therefore, no adjustment for multiple testing was performed. R version 4.3.2 was used for the statistical evaluation [26].

## Results

### Participant characteristics

In total, 606 individuals (average age: 28.96 ± 8.9 years, 326 women) were included in the study. Descriptive characteristics and perinatal parameters are presented in Table 1, stratified by the four GA groups. Significant disparities were observed across GA groups in education, income, occupation, and overall SES. The highest total SES was recorded in the GA ≥37 weeks group, with the lowest in the GA ≤ 28 weeks group. Additionally, significant variations were noted in ever being in a romantic relationship and childbearing, with the highest frequencies in the GA ≥ 37 weeks group. No differences were found in current relationship status or satisfaction. Regarding health-risk behaviours, significant differences were seen in alcohol consumption, but not in smoking (Table 2).

**Table 1.** Characteristics of the study sample (*N *= 606) stratified by gestational age (GA) groups

	Group 1	Group 2	Group 3	Group 4
	GA ≥ 37	GA 33–36	GA 29–32	GA ≤ 28
Number of participants	296	137	116	57
Age (years)	30.01 ± 9.4	29.47 ± 9.2	27.88 ± 8.0	24.51 ± 5.5
Women	156 (52.7%)	82 (59.9%)	60 (51.7%)	28 (49.1%)
Birth weight (BW), *M* ± SD	3436 ± 900	2068 ± 464	1500 ± 364	855 ± 215
BW < 1500 g (Yes)	1 (0.3%)	13 (9.5%)	55 (47.4%)	57 (100%)
BW < 1000 g (Yes)	0 (0%)	0 (0%)	10 (8.6%)	41 (71.9%)
Birth weight percentile, *M* ± SD	49.48 ± 36.88	25.25 ± 24.15	43.09 ± 26.42	37.60 ± 24.77
Gestational age (weeks), *M* ± SD	39.22 ± 1.49	34.31 ± 0.95	30.47 ± 1.16	26.32 ± 1.38
Maternal risk factors				
Preeclampsia (Yes)	31 (10.5%)	24 (17.5%)	16 (13.8%)	11 (19.3%)
Placental insufficiency (Yes)	13 (4.4%)	16 (11.7%)	3 (2.6%)	3 (5.3%)
Maternal smoking (Yes)	13 (4.4%)	8 (5.8%)	9 (7.8%)	8 (14.0%)
Haemolysis, Elevated Liver enzymes, and Low Platelet count syndrome (Yes)	1 (0.3%)	6 (4.4%)	2 (1.7%)	3 (5.3%)
Gestational diabetes (Yes)	5 (1.7%)	7 (5.1%)	2 (1.7%)	1 (1.8%)
Breastfeeding (Yes)	170 (57.4%)	75 (54.7%)	59 (50.9%)	25 (43.9%)

**Table 2.** Socio-economic factors, social relationships, and health-risk behaviours stratified by gestational age (GA)

	Group 1	Group 2	Group 3	Group 4	*P*-value
	GA ≥ 37	GA 33–36	GA 29–32	GA ≤ 28	
Number of participants	296	137	116	57	
Socio-economic factors					
Total socio-economic status score (range: 3–21 points)	13.41 ± 3.65	12.46 ± 3.72	12.54 ± 3.23	10.44 ± 3.29	<0.001
Education score (range: 1–7 points)	4.65 ± 1.65	4.38 ± 1.66	4.21 ± 1.50	3.34 ± 1.28	<0.001
Income score (range: 1–7 points)	5.48 ± 1.75	5.12 ± 1.85	5.40 ± 1.53	4.62 ± 1.96	0.004
Occupation score (range: 1–7 points)	3.28 ± 1.33	2.96 ± 1.16	2.92 ± 1.18	2.48 ± 0.97	<0.001
Romantic relationships and parenthood					
Ever been in a romantic relationship (Yes)	38/49 (77.6%)	14/27 (51.9%)	20/34 (58.8%)	11/24 (45.8%)	0.03
Present romantic relationship (Yes)	104/224 (46.4%)	49/97 (50.5%)	42/87 (48.3%)	20/54 (37.0%)	0.42
Satisfied with romantic relationship (Yes)	163/180 (90.6%)	73/84 (86.9%)	53/60 (88.3%)	19/21 (90.5%)	0.82
Having children (Yes)	72/296 (24.3%)	32/133 (24.1%)	18/110 (16.4%)	1/55 (1.8%)	0.001
Health-risk behaviours					
Current smoking (Yes)	50/296 (16.9%)	19/137 (13.9%)	15/116 (12.9%)	8/57 (14.0%)	0.71
≥1 glass of alcohol/week (Yes)	208/296 (70.3%)	88/137 (64.2%)	71/116 (61.2%)	22/57 (38.6%)	<0.001

*Note:* Percentages are calculated based on the number of participants with available data for each respective item. Due to missing data, the denominators vary between items.

### Univariable and multivariable regression analysis

Individuals born with a GA of ≤28 weeks demonstrated a lower odds ratio for having ever been in a romantic relationship (OR = 0.32; CI: 0.11–0.97; *P *= 0.05) (Table 3). The association with extremely preterm birth disappeared after including birth weight percentile groups (SGA and LGA) in model 2 (OR = 0.35; CI: 0.11–1.08; *P *= 0.07). In terms of having children, no association with the GA or birth weight percentile groups was evident. As for alcohol consumption, individuals with a GA of ≤ 28 weeks and 29–32 weeks were found to consume less alcohol (GA of ≤ 28 weeks: OR = 0.26; CI: 0.13–0.48, *P *< 0.001; 29–32 weeks: OR = 0.60, CI: 0.36–0.99; *P *= 0.05). Being born SGA was associated with lower alcohol consumption in adulthood (OR = 0.63; CI: 0.41–0.98; *P *= 0.04). No association was evident in terms of a present romantic relationship, relationship satisfaction, and current smoking.

**Table 3.** Association analyses of social relationships and health-risk behaviours for adults born preterm and full term (*N *= 606)

	Model 1		Model 2 With additional integration of birth weight percentile groups	
Social relationships				
Ever been in a relationship (Yes)	OR [95% confidence interval (CI)]	*P*-value	OR (95% CI)	*P*-value
Gestational age (GA) ≤ 28 weeks	0.32 (0.11–0.97)	0.05	0.35 (0.11–1.09)	0.07
GA 29–32 weeks	0.47 (0.18–1.26)	0.13	0.48 (0.17–1.39)	0.18
GA 33–36 weeks	0.35 (0.12–1.01)	0.05	0.42 (0.14–1.25)	0.12
Small for gestational age (SGA) (<10)			0.63 (0.25–1.56)	0.32
Large for gestational age (LGA) (>90)			2.40 (0.24–24.31)	0.46
Present romantic relationship (Yes)				
GA ≤ 28 weeks	0.81 (0.43–1.55)	0.53	0.94 (0.48–1.84)	0.87
GA 29–32 weeks	1.09 (0.65–1.83)	0.73	1.25 (0.72–2.15)	0.43
GA 33–36 weeks	1.15 (0.70–1.89)	0.58	1.31 (0.78–2.20)	0.30
SGA (<10)			1.03 (0.64–1.64)	0.91
LGA (>90)			1.91 (0.96–3.81)	0.07
Satisfied with romantic relationship (Yes)				
GA ≤ 28 weeks	1.03 (0.21–5.08)	0.97	1.54 (0.30–7.85)	0.60
GA 29–32 weeks	0.80 (0.31–2.06)	0.65	1.18 (0.44–3.18)	0.74
GA 33–36 weeks	0.70 (0.31–1.60)	0.40	0.91 (0.39–2.14)	0.83
SGA (<10)			2.15 (0.83–5.55)	0.11
LGA (>90)			3.52 (0.96–12.99)	0.06
Having children (Yes)				
GA ≤ 28 weeks	0.14 (0.02–1.20)	0.07	0.15 (0.02–1.34)	0.09
GA 29–32 weeks	0.93 (0.46–1.89)	0.84	1.00 (0.46–2.12)	0.99
GA 33–36 weeks	1.11 (0.60–2.08)	0.74	1.27 (0.66–2.46)	0.47
SGA (<10)			0.79 (0.40–1.52)	0.49
LGA (>90)			1.52 (0.71–3.24)	0.28
Health-risk behaviours				
At least 1 glass of alcohol per week (Yes)				
GA ≤ 28 weeks	0.27 (0.15–0.51)	<0.001	0.26 (0.13–0.49)	<0.001
GA 29–32 weeks	0.66 (0.42–1.06)	0.08	0.60 (0.36–0.99)	0.05
GA 33–36 weeks	0.86 (0.55–1.35)	0.52	0.88 (0.55–1.41)	0.60
SGA (<10)			0.63 (0.41–0.98)	0.04
LGA (>90)			0.95 (0.52–1.73)	0.88
Current smoking (Yes)				
GA ≤ 28 weeks	0.67 (0.28–1.58)	0.36	0.65 (0.27–1.57)	0.34
GA 29–32 weeks	0.70 (0.37–1.33)	0.28	0.68 (0.34–1.33)	0.25
GA 33–36 weeks	0.76 (0.42–1.37)	0.36	0.75 (0.40–1.38)	0.35
SGA (<10)			0.91 (0.52–1.60)	0.74
LGA (>90)			0.91 (0.45–1.83)	0.79

Both models are adjusted for age, gender, and socio-economic status.

*Note*: A GA of ≥37 weeks was used as the reference category for the GA groups. Appropriate for GA (AGA, 10th–90th percentile) was used as the reference category for SGA and LGA individuals.

Analyses using continuous measures for GA deficits (weeks) and birth weight percentiles additionally show a continued association between GA deficit and ever having been in a romantic relationship (OR = 0.92; CI: 0.85–1.00; *P *= 0.05) (Table S1). Having children (yes) in adulthood was examined in a sensitivity analysis only for those aged 35 and older in the cohort (Table S2). The analysis revealed an association between a greater GA deficit and a lower likelihood of having children in adulthood (OR = 0.89; CI: 0.81–0.98; *P *= 0.02).

In examining socio-economic outcomes, the results indicate that lower GA (≤28 weeks and 29–32 weeks) is significantly associated with lower educational outcomes (Table 4). Regarding occupation scores, all degrees of prematurity were negatively associated with occupation scores. Furthermore, our findings show that in terms of income scores, a GA of 28 weeks or less and 33-36 weeks are significantly associated with lower income scores. The SES was negatively associated with a lower GA of ≤ 28 weeks and 33–36 weeks (extremely preterm: β= −2.25; 95% CI: −3.22 to −1.27; *P *< 0.001; moderately preterm: β = −1.04; 95% CI: −1.73 to −0.35; *P *= 0.003) (Fig. S2). Foetal growth restriction and foetal overgrowth showed no association with SES or socio-economic categories (Table 4). The analysis of the same model with continuous parameters also shows an association between GA deficit and lower education score, occupation score, income score, and lower overall SES status. Likewise, the birth weight percentile is associated with a lower income score and overall SES (Table S3). In sensitivity analyses additionally adjusting for maternal education score, the associations between extreme prematurity and lower education and occupation outcomes remained present (Table S4). The negative association with the overall SES score also persisted. The associations with income scores were no longer statistically significant after this additional adjustment (Table S4).

**Table 4.** Association analyses of the socio-economic parameters for adults born preterm and full term (*n* = 606)

	Model 1		Model 2	
Education score	Estimate [95% confidence interval (CI)]	*P*-value	Estimate (95% CI)	*P*-value
Gestational age (GA) ≤ 28 weeks	−0.98(−1.41 to −0.54)	<0.001	−1.05(−1.50 to −0.60)	<0.001
GA 29–32 weeks	−0.31(−0.63 to 0.01)	0.06	−0.41(−0.75 to −0.06)	0.02
GA 33–36 weeks	−0.27(−0.58 to 0.03)	0.08	−0.29(−0.61 to 0.02)	0.07
Small for gestational age (SGA) (<10)			−0.28(−0.58 to 0.02)	0.07
Large for gestational age (LGA) (>90)			−0.19(−0.58 to 0.20)	0.35
Occupation score				
GA ≤ 28 weeks	−0.42(−0.74 to −0.11)	0.01	−0.48(−0.80 to −0.16)	0.004
GA 29–32 weeks	−0.22(−0.45 to 0.02)	0.07	−0.27(−0.52 to −0.02)	0.03
GA 33–36 weeks	−0.29(−0.51 to −0.07)	0.01	−0.33(−0.56 to −0.10)	0.005
SGA (<10)			−0.06(−0.28 to 0.15)	0.56
LGA (>90)			−0.19(−0.47 to 0.09)	0.18
Income score				
GA ≤ 28 weeks	−0.68(−1.18 to −0.18)	0.01	−0.72(−1.23 to −0.20)	0.01
GA 29–32 weeks	−0.006(−0.38 to 0.37)	0.98	−0.01(−0.41 to 0.38)	0.94
GA 33–36 weeks	−0.34(−0.69 to 0.01)	0.06	−0.41(−0.78 to −0.05)	0.03
SGA (<10)			0.27(−0.07 to 0.62)	0.12
LGA (>90)			−0.24(−0.69 to 0.21)	0.30
Socio−economic status score (SES)				
GA ≤ 28 weeks	−2.08(−3.03 to −1.14)	<0.001	−2.25(−3.22 to −1.27)	<0.001
GA 29–32 weeks	−0.53(−1.24 to 0.17)	0.14	−0.69(−1.45 to 0.06)	0.07
GA 33–36 weeks	−0.90(−1.57 to −0.24)	0.01	−1.04(−1.73 to −0.35)	0.003
SGA (<10)			−0.07(−0.72 to 0.58)	0.83
LGA (>90)			−0.62(−1.47 to 0.23)	0.16

Adjusted for age at study examination and gender.

To further explore gender-related effects beyond the main analyses (which were adjusted for gender), we conducted additional models that included gender as a main effect and tested interaction terms between gender and GA deficit (Tables S5–S7). In these analyses, gender was significantly associated with several outcomes: women had higher education scores but lower income scores and reported lower alcohol consumption compared with men. Women were also more likely to report having children and to be currently in a romantic relationship. Regarding effect modification, a significant interaction between GA deficit and gender was observed for the income score (*P* = 0.02). In reparametrized models with women as the reference category, GA deficit was not associated with income score in women (β = −0.009; 95% CI: −0.05 to 0.03; *P* = 0.66), whereas the corresponding association was negative in men (β = −0.08). For overall SES, GA deficit was negatively associated with SES in women (β = −0.09; 95% CI: −0.17 to −0.01; *P* = 0.02), while the interaction term indicated a trend toward a stronger inverse association in men (*P* = 0.09). No significant interactions were found for the remaining outcomes.

## Discussion

This study examined the long-term associations of prematurity and foetal growth restriction with adult social relationships, health-risk behaviours, and socio-economic outcomes. Prematurity showed the most consistent associations across domains, whereas birth weight percentile contributed less to the observed differences.

Our findings indicate descriptively that study participants with a GA of ≤ 28 weeks were less likely to have ever been in a romantic relationship. The multivariable analysis, with the usage of continuous parameters, found that lower GA and lower birth weight percentile are associated with a decreased likelihood of ever being in a romantic relationship. Similar findings have been observed in other studies [14]. Individuals born full term were confirmed to be more likely to show a tendency toward extraversion, meaning that they tend to be more sociable and actively seek out social relationships, while individuals born preterm or with an LBW are more likely to tend towards introversion and thus be less socially engaged [27]. Another plausible explanation could be the immaturity of intrauterine development related to stress processing or disruptions in attachment development and the formation of primary trust [28]. Depending on the level of parental care in neonatal intensive care units, factors such as early separation from parents, disturbances in bonding [29], or overprotection by parents [30] might play a significant role in shaping these individuals’ ability to form social relationships later in life.

Regarding parenthood, associations were not evident in the full sample. However, in the sensitivity analysis restricted to participants aged 35 years and older, greater GA deficit was associated with a lower likelihood of having children. This aligns with prior work reporting reduced parenthood rates among adults born preterm or with LBW [14]. This pattern may partly reflect reduced partnership formation and socio-economic constraints relevant to family planning.

For health-risk behaviours, being born extremely or very preterm was associated with lower alcohol consumption, and foetal growth restriction was also associated with lower alcohol consumption. Other studies have also established that a lower GA or LBW is associated with less risk-taking behaviour [15, 31]. In a study involving individuals born with a very LBW (*n* = 242) with a mean GA of 29.7 weeks, the individuals displayed better health behaviour, with less alcohol consumption in adulthood compared to 233 controls born with a normal birth weight. There was no differentiation between GA and birth weight as a driving factor [31].

In terms of socio-economic outcomes, poorer scores in dimensions such as education, employment, and income were observed, particularly among those born extremely and very preterm. A study of 12-year-old children, based on the examination of 860 332 individuals born in the Netherlands from 1999 to 2006 at 25–42 weeks gestation, also found that in terms of education, children with a higher GA had a better school performance than children with a lower GA [32]. The authors hypothesize that preterm birth, and the consequent interruption of brain maturation in the womb, could be a contributing factor to poorer academic performance in individuals with a low GA [32]. If not addressed, such early deficits can have a sustained impact on an individual’s educational and career trajectory. A further analysis based on Swedish registers for the 1982–94 birth cohorts also reports that extremely premature babies (<28 weeks) had poorer school grades than full term babies at the age of 16. The birth weight percentiles were not included in the analysis [33]. We confirm these findings and additionally expand on the available evidence base as we were able to demonstrate that adults with a GA ≤ 28 weeks and of 29–32 weeks have a lower educational status, while foetal growth restriction and foetal overgrowth were not associated with the educational score. This finding allows us to distinguish the impact of different levels of prematurity from foetal growth restriction and identify prematurity as a key factor contributing to poorer educational outcomes. This may point to the high relevance of providing individuals born preterm with special support at school so that they can achieve the same educational opportunities and thus later career opportunities as individuals born full term.

In addition, a lower income score was also found for individuals with a low GA ≤ 28 weeks and 33–36 weeks. However, this association was no longer statistically significant after additional adjustment for maternal education score in sensitivity analyses. It should be noted, however, that this analysis had reduced statistical power due to the smaller sample with available maternal education (*n* = 257).

Another study of adults aged 29–36 years showed that individuals born with an extremely LBW (*n* = 100) earned on average $20 000/year less than control individuals born full term (*n* = 89). There was no differentiation made between GA and birth weight percentile [34]. Moreover, our findings show an association between lower occupational points with prematurity (GA ≤ 28 weeks, 29–32 weeks, and 33–36 weeks). Other studies in young adults born preterm or/and with very LBW (without differentiation) also yield poorer occupational outcomes in individuals born preterm [19, 34].

We additionally examined gender-related effects and potential effect modification by gender. In these analyses, gender was significantly associated with several outcomes: women had higher education scores but lower income scores and reported lower alcohol consumption compared with men. Women were also more likely to report having children and to be currently in a romantic relationship. Gender differences in educational attainment, income, and alcohol consumption have been consistently reported in prior studies [35, 36]. In our data, the interaction for income indicates that a larger GA deficit was linked to lower income scores primarily in men. For overall SES, the interaction term likewise suggested a stronger inverse association in men. One plausible explanation is that income in women is less tightly coupled to individual health-related capacity because it is more strongly shaped by part-time work and employment interruptions, whereas men’s income more often reflects continuous labour-market participation and performance [37]. However, these interaction findings were exploratory and should not be overinterpreted.

Overall, in our study, individuals born preterm display poorer scores with lower GA in all three dimensions of SES as well as in overall SES. Possible reasons for this association are developmental deficits often observed in individuals born preterm [38]. Premature birth can disrupt the maturation of critical neurological pathways, which in turn impairs cognitive, motor, and social development [39]. These developmental deficits can affect long-term socio-economic outcomes and opportunities. Another reason could be that premature births occur in families with a low SES [40]. Our analysis with continuous parameters also showed a correlation between lower birth weight percentile and lower SES. These factors could explain the observed associations. Our data cannot exclude the possibility that parental SES influences both the risk of preterm birth and the child’s SES.

Further research is necessary to better comprehend these relationships and to develop targeted interventions that can mitigate the long-term socio-economic impacts of prematurity and foetal overgrowth.

### Strengths and limitations

This study has several limitations. First, it was conducted as a single-centre, hospital-based cohort study with all participants recruited from one clinic. Therefore, local clinical practices, referral patterns, and characteristics of the clinic’s catchment area (e.g., socio-demographic composition and regional healthcare structures) may have influenced both neonatal management and later-life outcomes. As a result, the external validity of the findings may be limited, and replication in multicentre cohorts and in other healthcare settings is warranted. Some former newborns could not be reached, and others chose not to participate, which introduces the possibility of selection bias. Moreover, most participants were Caucasians, so the findings cannot be generalized to other ethnic groups. Another important limitation relates to the generalizability of our findings. Advances in neonatal care, such as the introduction of antenatal corticosteroids, magnesium sulphate administration for neuroprotection, and surfactant therapy, have significantly improved the survival and long-term outcomes of preterm infants over recent decades. Since our cohort includes individuals born between 1969 and 2002, these advances were not uniformly available during the entire study period. Therefore, the psychosocial and socio-economic outcomes observed in this study may not fully reflect the experiences of more recently born preterm individuals. Caution is thus warranted when generalizing the results to current or future cohorts of preterm births. For some relationship-related outcomes, the number of available responses was lower than the overall sample size. As a result, estimates for these outcomes are based on smaller subsets and should be interpreted in light of the reduced precision. Moreover, parental SES was not available for the cohort. Therefore, residual confounding by intergenerational socio-economic factors cannot be excluded. Sensitivity analyses adjusting for maternal education in a subset of participants demonstrated consistent results for education, occupation, and overall SES.

Despite these limitations, a major strength of this study is the detailed differentiation between multiple degrees of prematurity (extremely preterm, very preterm, and moderately preterm) and the separate assessment of foetal growth restriction. This distinction allows for a more nuanced analysis of how varying levels of GA and birth weight independently relate to psychosocial and socio-economic outcomes in adulthood. Furthermore, the study benefits from a comparatively large cohort of adults born preterm alongside a substantial control group.

## Conclusion

In this cohort, prematurity was associated with lower alcohol consumption, a lower likelihood of ever having been in a romantic relationship, and lower SES in adulthood, whereas foetal growth restriction showed more limited associations. In participants aged ≥35 years, lower GA was additionally associated with a lower likelihood of parenthood. Overall, the findings suggest that prematurity, rather than foetal growth restriction, is the primary early-life factor linked to adverse socio-economic outcomes later in life.

## Supplementary Material

ckag105_Supplementary_Data

## Data Availability

Data are available upon reasonable request. The analysis presents the clinical data of a cohort. This project constitutes a major scientific effort with high methodological standards and detailed guidelines for analysis and publication to ensure scientific analyses are on the highest level, therefore, data are not made available for the scientific community outside the established and controlled workflows and algorithms. To meet the general idea of verification and reproducibility of scientific findings, we offer access to data at the local database upon request at any time. Interested researchers should make their requests to the coordinating PI of the GPS (Achim Fieß; achim.fiess@unimedizin-mainz.de). More detailed contact information is available at the homepages of the UM (www.unimedizin-mainz.de).
