# Supplementary material for: Relevance of prematurity and foetal growth restriction for romantic relationships, health-risk behaviours, and socio-economic outcomes in adulthood
Source: Eur J Public Health. 2026 Jul 14;36(4):ckag105. doi: 10.1093/eurpub/ckag105 (PMC13368824; doi:10.1093/eurpub/ckag105)
Supplement: ckag105_Supplementary_Data [file ckag105_supplementary_data.zip › ejph-2025-04-om-0285-File010.docx]

**Table S3.** Association analyses of the socioeconomic parameters for adults born preterm and full term (n = 606), with usage of continuous parameters.

|  | Model 1 | | | Model 2 | |
| --- | --- | --- | --- | --- | --- |
| **Education score** | Estimate  (95% CI) | | p-value | Estimate  (95% CI) | p-value |
| Gestational age deficit (weeks) | -0.06  (-0.09, -0.04) | | <0.001 | -0.06  (-0.09, -0.04) | <0.001 |
| BW Percentile |  | |  | -0.001  (-0.005, 0.003) | 0.62 |
| **Occupation score** | | | |  |  |
| Gestational age deficit (weeks) | -0.03  (-0.05, -0.01) | 0.003 | | -0.03  (-0.05, -0.01) | 0.001 |
| BW Percentile |  |  | | -0.002  (-0.004, 0.001) | 0.12 |
| **Income score** | | | |  |  |
| Gestational age deficit (weeks) | -0.04  (-0.07, -0.006) | 0.02 | | -0.04  (-0.07, -0.01) | 0.006 |
| BW Percentile |  |  | | -0.005  (-0.01, -0.001) | 0.03 |
| **Socioeconomic status score (SES)** | | | |  |  |
| Gestational age deficit (weeks) | -0.13  (-0.18, -0.07) | <0.001 | | -0.14  (-0.19, -0.07) | <0.001 |
| BW Percentile |  |  | | -0.01  (-0.02, -0.00) | 0.05 |

* Gestational age deficit represents the number of weeks by which the gestation is shorter than the standard full term pregnancy of 40 weeks.
