# Supplementary material for: Relevance of prematurity and foetal growth restriction for romantic relationships, health-risk behaviours, and socio-economic outcomes in adulthood
Source: Eur J Public Health. 2026 Jul 14;36(4):ckag105. doi: 10.1093/eurpub/ckag105 (PMC13368824; doi:10.1093/eurpub/ckag105)
Supplement: ckag105_Supplementary_Data [file ckag105_supplementary_data.zip › ejph-2025-04-om-0285-File011.docx]

**Table S4**. Sensitivity analyses of the socioeconomic parameters for adults born preterm and full term (n = 606). Adjusted for age at study examination, gender and maternal education score.

|  | Model 1 | | | Model 2 | |
| --- | --- | --- | --- | --- | --- |
| **Education score** | Estimate  (95% CI) | | p-value | Estimate  (95% CI) | p-value |
| GA ≤ 28 weeks | -1.12  (-1.72, -0.51) | | <0.001 | -1.12  (-1.76, -0.49) | <0.001 |
| GA 29–32 weeks | -0.29  (-0.76, 0.18) | | 0.23 | -0.29  (-0.79, 0.22) | 0.26 |
| GA 33-36 weeks | -0.38  (-0.83, 0.07) | | 0.10 | -0.42  (-0.89, 0.05) | 0.08 |
| SGA (<10) |  | |  | 0.14  (-0.31, 0.45) | 0.55 |
| LGA (>90) |  | |  | -0.07  (-0.58, 0.45) | 0.81 |
| **Occupation score** | | | |  |  |
| GA ≤ 28 weeks | -0.46  (-0.94, 0.01) | 0.06 | | -0.54  (-1.04, -0.05) | 0.03 |
| GA 29–32 weeks | -0.24  (-0.61, 0.13) | 0.20 | | -0.31  (-0.70, 0.09) | 0.13 |
| GA 33-36 weeks | -0.21  (-0.56, 0.15) | 0.25 | | -0.32  (-0.69, 0.05) | 0.09 |
| SGA (<10) |  |  | | 0.16  (-0.19, 0.51) | 0.37 |
| LGA (>90) |  |  | | -0.31  (-0.71, 0.09) | 0.13 |
| **Income score** | | | |  |  |
| GA ≤ 28 weeks | -0.57  (-1.34, 0.20) | 0.15 | | -0.72  (-1.52, 0.08) | 0.08 |
| GA 29–32 weeks | 0.02  (-0.57, 0.61) | 0.95 | | -0.11  (-0.75, 0.52) | 0.72 |
| GA 33-36 weeks | -0.42  (-0.99, 0.15) | 0.15 | | -0.58  (-1.17, 0.02) | 0.06 |
| SGA (<10) |  |  | | 0.09  (-0.48, 0.65) | 0.76 |
| LGA (>90) |  |  | | -0.51  (-1.16, 0.14) | 0.12 |
| **Socioeconomic status score (SES)** | | | |  |  |
| GA ≤ 28 weeks | -2.15  (-3.54, -0.76) | 0.003 | | -2.39  (-3.85, -0.94) | 0.001 |
| GA 29–32 weeks | -0.51  (-1.59, 0.57) | 0.35 | | -0.71  (-1.87, 0.44) | 0.23 |
| GA 33-36 weeks | -1.01  (-2.04, 0.03) | 0.06 | | -1.32  (-2.39, -0.24) | 0.02 |
| SGA (<10) |  |  | | 0.39  (-0.64, 1.41) | 0.46 |
| LGA (>90) |  |  | | -0.89  (-2.07, 0.29) | 0.15 |
