# Supplementary material for: Relevance of prematurity and foetal growth restriction for romantic relationships, health-risk behaviours, and socio-economic outcomes in adulthood
Source: Eur J Public Health. 2026 Jul 14;36(4):ckag105. doi: 10.1093/eurpub/ckag105 (PMC13368824; doi:10.1093/eurpub/ckag105)
Supplement: ckag105_Supplementary_Data [file ckag105_supplementary_data.zip › ejph-2025-04-om-0285-File012.docx]

**Table S5**. Association analyses of social relationships and health-risk behaviors in adults born preterm and full term, including interaction terms with gender (n = 606). Adjusted for age and socioeconomic status.

| **Social relationships** | | |
| --- | --- | --- |
| **Ever been in a romantic relationship** | OR  (95% CI) | p-value |
| Gestational age deficit (weeks) | 0.99  (0.96, 1.01) | 0.21 |
| BW percentiles | 1.00  (1.00, 1.01) | 0.04 |
| Gender (female) | 0.94  (0.73, 1.23) | 0.66 |
| Gestational age deficit (weeks)* Gender (female) | 0.99  (0.96, 1.03) | 0.74 |
| **Present romantic relationship** | | |
| Gestational age deficit (weeks) | 1.03  (0.97, 1.10) | 0.30 |
| BW percentiles | 1.00  (0.99, 1.01) | 0.78 |
| Gender (female) | 2.59  (1.46, 4.64) | 0.001 |
| Gestational age deficit (weeks)* Gender (female) | 0.93  (0.86, 1.01) | 0.08 |
| **Satisfied with romantic relationship** | | |
| Gestational age deficit (weeks) | 1.03  (0.91, 1.17) | 0.69 |
| BW percentiles | 1.00  (0.99, 1.01) | 0.90 |
| Gender (female) | 1.94  (0.71, 5.46) | 0.20 |
| Gestational age deficit (weeks)* Gender (female) | 0.92  (0.78, 1.07) | 0.29 |
| **Having children (yes)** | | |
| Gestational age deficit (weeks) | 0.99  (0.90, 1.09) | 0.84 |
| BW percentiles | 1.00  (1.00, 1.01) | 0.36 |
| Gender (female) | 2.10  (1.04, 4.30) | 0.04 |
| Gestational age deficit (weeks)* Gender (female) | 0.92  (0.81, 1.05) | 0.24 |
| **Health-risk behaviors** | | |
| **Smoking** | | |
| Gestational age deficit (weeks) | 0.97  (0.91, 1.04) | 0.43 |
| BW Percentile | 1.00  (1.00, 1.01) | 0.18 |
| Gender (female) | 0.56  (0.30, 1.05) | 0.07 |
| Gestational age deficit (weeks)* Gender (female) | 0.96  (0.86, 1.06) | 0.44 |
| **At least 1 Glass of alcohol per week** | | |
| Gestational age deficit (weeks) | 0.99  (0.97, 1.00) | 0.01 |
| BW Percentile | 1.00  (1.00, 1.00) | 0.42 |
| Gender (female) | 0.82  (0.74, 0.91) | <0.001 |
| Gestational age deficit (weeks)* Gender (female) | 1.00  (0.98, 1.02) | 0.92 |

* Gestational age deficit represents the number of weeks by which the gestation is shorter than the standard full term pregnancy of 40 weeks.
