# Supplementary material for: Relevance of prematurity and foetal growth restriction for romantic relationships, health-risk behaviours, and socio-economic outcomes in adulthood
Source: Eur J Public Health. 2026 Jul 14;36(4):ckag105. doi: 10.1093/eurpub/ckag105 (PMC13368824; doi:10.1093/eurpub/ckag105)
Supplement: ckag105_Supplementary_Data [file ckag105_supplementary_data.zip › ejph-2025-04-om-0285-File014.docx]

**Table S7.** Association analyses of income and overall socioeconomic status for adults born preterm and full term (n = 606), including interaction terms with gender. Adjusted for age.

| **Income score** | Estimate  (95% CI) | p-value |
| --- | --- | --- |
| Gestational age deficit (weeks) | -0.009  (-0.05, 0.03) | 0.66 |
| BW Percentile | -0.005  (-0.01, -0.001) | 0.01 |
| Gender (male) | 0.44  (0.04, 0.84) | 0.03 |
| Gestational age deficit (weeks)* Gender (male) | -0.07  (-0.13, -0.011) | 0.02 |
| **Socioeconomic status score (SES)** | | |
| Gestational age deficit (weeks) | -0.09  (-0.17, -0.01) | 0.02 |
| BW Percentile | -0.009  (-0.02, -0.001) | 0.04 |
| Gender (male) | 0.03  (-0.72, 0.79) | 0.93 |
| Gestational age deficit (weeks)* Gender (male) | -0.10  (-0.21, 0.02) | 0.09 |

* Gestational age deficit represents the number of weeks by which the gestation is shorter than the standard full term pregnancy of 40 weeks. In this table, women were the reference category for gender.
