# Supplementary material for: Relevance of prematurity and foetal growth restriction for romantic relationships, health-risk behaviours, and socio-economic outcomes in adulthood
Source: Eur J Public Health. 2026 Jul 14;36(4):ckag105. doi: 10.1093/eurpub/ckag105 (PMC13368824; doi:10.1093/eurpub/ckag105)
Supplement: ckag105_Supplementary_Data [file ckag105_supplementary_data.zip › ejph-2025-04-om-0285-File013.docx]

**Table S6.** Association analyses of the socioeconomic parameters for adults born preterm and full term (n = 606), including interaction terms with gender. Adjusted for age.

| **Education score** | Estimate  (95% CI) | p-value |
| --- | --- | --- |
| Gestational age deficit (weeks) | -0.07  (-0.11, -0.03) | <0.001 |
| BW Percentile | -0.001  (-0.005, 0.003) | 0.58 |
| Gender (female) | 0.36  (0.01, 0.71) | 0.04 |
| Gestational age deficit (weeks)* Gender (female) | 0.02  (-0.04, 0.07) | 0.56 |
| **Occupation score** | | |
| Gestational age deficit (weeks) | -0.04  (-0.06, -0.01) | 0.01 |
| BW Percentile | -0.002  (-0.004, 0.001) | 0.11 |
| Gender (female) | 0.05  (-0.20, 0.30) | 0.69 |
| Gestational age deficit (weeks)* Gender (female) | 0.01  (-0.03, 0.05) | 0.61 |
| **Income score** | | |
| Gestational age deficit (weeks) | -0.08  (-0.12, -0.04) | <0.001 |
| BW Percentile | -0.005  (-0.01, -0.001) | 0.01 |
| Gender (female) | -0.44  (-0.84, -0.04) | 0.03 |
| Gestational age deficit (weeks)* Gender (female) | 0.07  (0.01, 0.13) | 0.02 |
| **Socioeconomic status score (SES)** | | |
| Gestational age deficit (weeks) | -0.19  (-0.27, -0.11) | <0.001 |
| BW Percentile | -0.009  (-0.02, -0.001) | 0.04 |
| Gender (female) | -0.03  (-0.79, 0.72) | 0.93 |
| Gestational age deficit (weeks)* Gender (female) | 0.10  (-0.02, 0.21) | 0.09 |

* Gestational age deficit represents the number of weeks by which the gestation is shorter than the standard full term pregnancy of 40 weeks. In this table, men were the reference category for gender.
