# Supplementary material for: Relevance of prematurity and foetal growth restriction for romantic relationships, health-risk behaviours, and socio-economic outcomes in adulthood
Source: Eur J Public Health. 2026 Jul 14;36(4):ckag105. doi: 10.1093/eurpub/ckag105 (PMC13368824; doi:10.1093/eurpub/ckag105)
Supplement: ckag105_Supplementary_Data [file ckag105_supplementary_data.zip › ejph-2025-04-om-0285-File008.docx]

**Table S1**. Association analyses of social relationships and health-risk behaviors for adults born preterm and full term, with usage of continuous parameters (n=606).

|  | Model 1 | | Model 2 | |
| --- | --- | --- | --- | --- |
| **Social relationships** | | | | |
| **Ever been in a romantic relationship** | OR  (95% CI) | p-value | OR  (95% CI) | p-value |
| Gestational age deficit (weeks) | 0.92  (0.85, 0.99) | 0.04 | 0.92  (0.85, 1.00) | 0.05 |
| BW percentiles |  |  | 1.01  (1.00, 1.03) | 0.04 |
| **Present romantic relationship** | | | | |
| Gestational age deficit (weeks) | 0.99  (0.95, 1.03) | 0.68 | 0.99  (0.95, 1.03) | 0.70 |
| BW percentiles |  |  | 1.00  (0.99, 1.01) | 0.91 |
| **Satisfied with romantic relationship** | | | | |
| Gestational age deficit (weeks) | 0.98  (0.90, 1.06) | 0.62 | 0.98  (0.90, 1.06) | 0.62 |
| BW percentiles |  |  | 1.00  (0.99, 1.01) | 0.94 |
| **Having children** |  |  |  |  |
| Gestational age deficit (weeks) | 0.94  (0.88, 1.00) | 0.07 | 0.95  (0.89, 1.02) | 0.13 |
| BW percentiles |  |  | 1.00  (0.99, 1.01) | 0.44 |
| **Health-risk behaviors** | | | | |
| **Smoking** | | | | |
| Gestational age deficit (weeks) | 0.95  (0.90, 1.00) | 0.05 | 0.96  (0.91, 1.01) | 0.13 |
| BW Percentile |  |  | 1.00  (1.00, 1.01) | 0.19 |
| **At least 1 Glass of alcohol per week** | | | | |
| Gestational age deficit (weeks) | 0.93  (0.89, 0.96) | <0.001 | 0.93  (0.90, 0.97) | <0.001 |
| BW Percentile |  |  | 1.00  (1.00, 1.01) | 0.33 |

* Gestational age deficit represents the number of weeks by which the gestation is shorter than the standard full term pregnancy of 40 weeks.
