# Supplementary material for: Relevance of prematurity and foetal growth restriction for romantic relationships, health-risk behaviours, and socio-economic outcomes in adulthood
Source: Eur J Public Health. 2026 Jul 14;36(4):ckag105. doi: 10.1093/eurpub/ckag105 (PMC13368824; doi:10.1093/eurpub/ckag105)
Supplement: ckag105_Supplementary_Data [file ckag105_supplementary_data.zip › ejph-2025-04-om-0285-File009.docx]

**Table S2.** Sensitivity analysis for having children in adulthood. Participants under 35 years of age were excluded (n = 146).

|  | Model 1 | | Model 2 | |
| --- | --- | --- | --- | --- |
| **Social relationships** | | | | |
| **Having children** | OR  (95% CI) | p-value | OR  (95% CI) | p-value |
| Gestational age deficit (weeks) | 0.89  (0.80, 0.98) | 0.02 | 0.89  (0.81, 0.98) | 0.02 |
| BW percentiles |  |  | 1.00  (0.99, 1.01) | 0.92 |

* Gestational age deficit represents the number of weeks by which the gestation is shorter than the standard full term pregnancy of 40 weeks.
