# Supplementary figures and images for: Relevance of prematurity and foetal growth restriction for romantic relationships, health-risk behaviours, and socio-economic outcomes in adulthood
Source: Eur J Public Health. 2026 Jul 14;36(4):ckag105. doi: 10.1093/eurpub/ckag105 (PMC13368824; doi:10.1093/eurpub/ckag105)

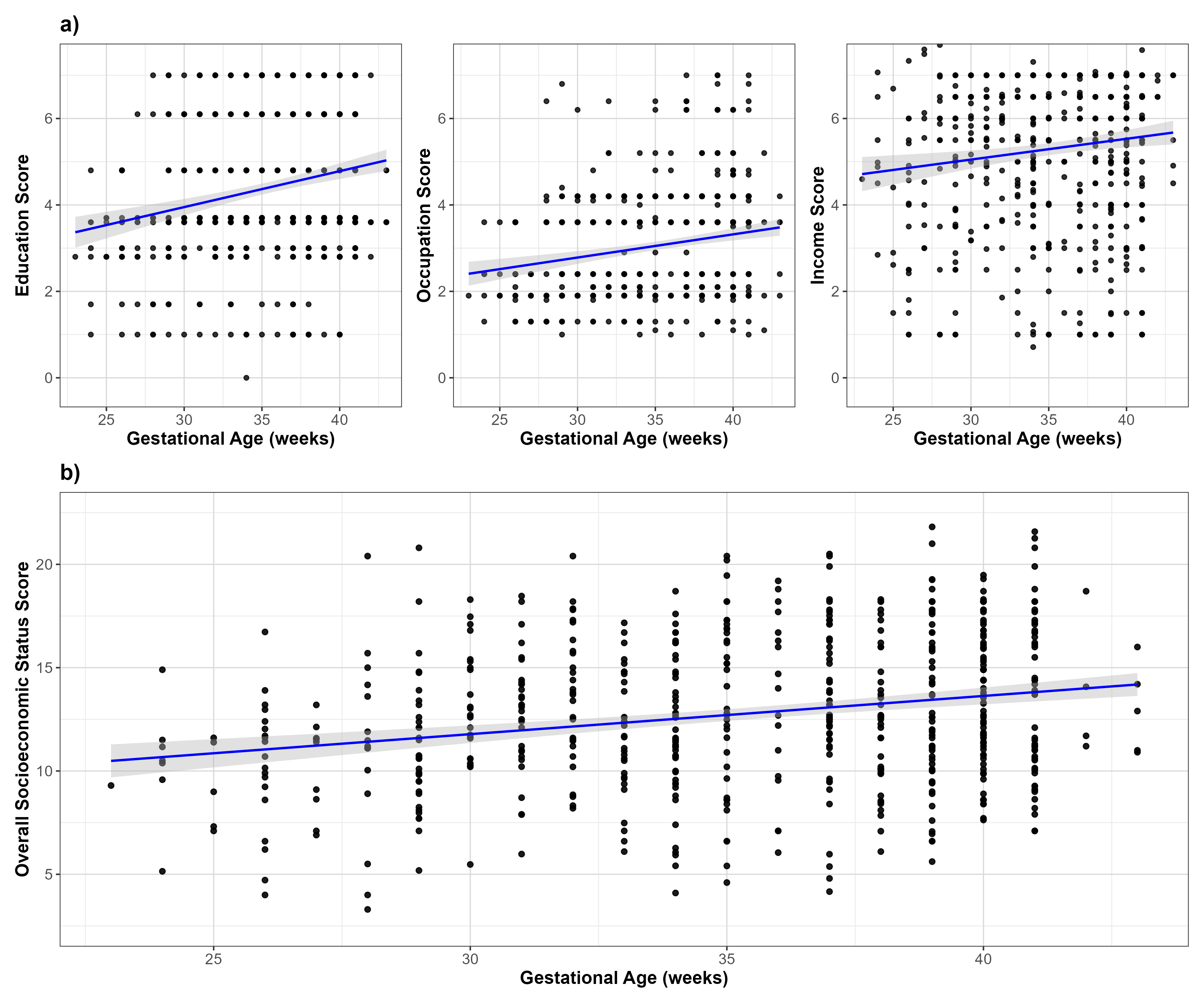

Supplement: ckag105_Supplementary_Data [file ckag105_supplementary_data.zip › ejph-2025-04-om-0285-File007.tiff]

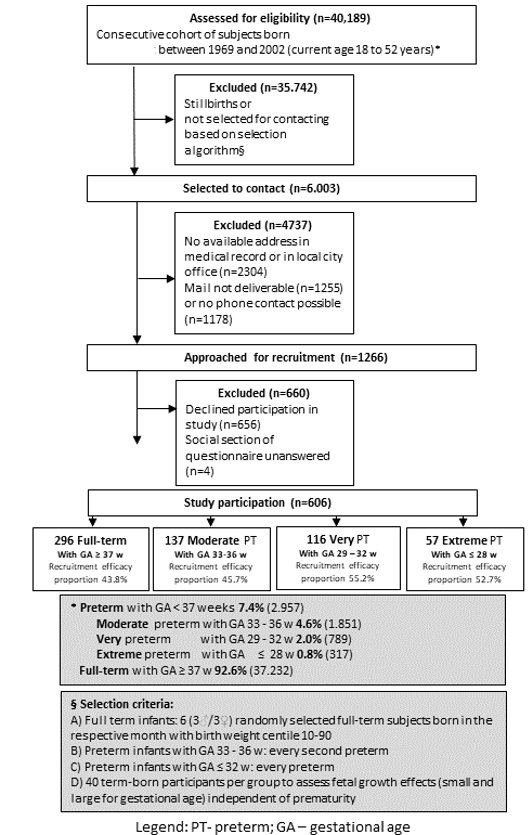

Supplement: ckag105_Supplementary_Data [file ckag105_supplementary_data.zip › ejph-2025-04-om-0285-File006.tif]
